# Clinical Variation of *Plasmodium falciparum eba-175*, *ama-1*, and *msp-3* Genotypes in Young Children Living in a Seasonally High Malaria Transmission Setting in Burkina Faso

**DOI:** 10.1155/2015/985651

**Published:** 2015-11-08

**Authors:** Issiaka Soulama, Samuel S. Sermé, Edith C. Bougouma, Amidou Diarra, Alfred B. Tiono, Alphonse Ouedraogo, Amadou T. Konate, Issa Nebie, Sodiomon B. Sirima

**Affiliations:** ^1^Centre National de Recherche et de Formation sur le Paludisme (CNRFP), 01 BP 2208, Ouagadougou 01, Burkina Faso; ^2^Groupe de Recherche Action en Santé, 06 BP 10248, Ouagadougou 06, Burkina Faso

## Abstract

The association between *P. falciparum eba-175*, *ama-1*, and *msp-3 *polymorphism in the pathogenicity of malaria disease was investigated. We therefore compared the prevalence of different alleles between symptomatic and asymptomatic malarial children under five years of age living in Burkina Faso. Blood filter papers were collected during the 2008 malaria transmission season from 228 symptomatic and 199 asymptomatic children under five years of age. All patients were living in the rural area of Saponé at about 50 km from Ouagadougou, the capital city of Burkina Faso. *P. falciparum *parasite DNA was extracted using QIAGEN kits and the alleles diversity was assessed by a nested PCR. PCR products were then digested by restriction enzymes based on already described polymorphic regions of the *eba-175*, *ama-1*, and *msp-3 *genes. The individual alleles *eba-175_FCR3* and *msp-3_K1* frequencies were statistically higher (*p* < 0.0001) in the asymptomatic group compared to the symptomatic ones. No statistically significant difference was noted in the prevalence of *ama-1*-3D7, *ama-1*-K1, and *ama-1*-HB3 genotypes between the two groups (*p* > 0.05). The comparative analysis of *P. falciparum *genotypes indicated that the polymorphism in *eba*-*175* and *msp-3 *genotypes varied between asymptomatic and symptomatic clinical groups and may contribute to the pathogenesis of malaria.

## 1. Introduction

According to the latest WHO malaria report [1], there were in 2014 about 197 million malaria cases worldwide and an estimated 584 000 deaths, mostly among African children. Unfortunately, although active implementation of malaria control strategies including global deployment of artemisinin-based combination therapies (ACTs) is underway, malaria remains a major public health concern, especially in sub-Saharan Africa. The genetic complexity of malaria parasites represents one of the main obstacles to the effective control of the disease. The relationship between the parasite genotype and the clinical presentation of malaria has already been reported elsewhere [2–4]. Erythrocytes binding antigen-175 (EBA-175), apical membrane antigen-1 (AMA-1), and merozoite surface protein 3 (MSP-3) are merozoite surface antigens thought to play a key role in red blood cell invasion by the parasite [5–8]. An association between both* ama-1* and* msp-3* genotypes and protection from malaria has been previously reported in earlier seroepidemiological studies [9–11]. In addition,* eba-175* C-genotypes were found to be associated with fatal outcome in severe malaria [12]. In 2001, Policy and Conway [13] revealed the allele specific immunity of* P. falciparum msp-3* K1 allelic form. Based on the above clinical associations and the polymorphism in* eba-175* [12],* msp-3* [14], and* ama-1* [15, 16], we believe that the assessment of the clinical variation of* eba-175*,* msp-3*, and* ama-1* haplotypes in symptomatic and asymptomatic children living in a malaria endemic and specifically in seasonal areas is of scientific interest. Indeed, the results from this current study could help to improve our understanding of the role, if any, of* P. falciparum* genotype diversity in malaria pathogenicity. Moreover, data generated from this study might be relevant to vaccine studies in the context of EBA-175, AMA-1, and MSP-3 based malaria vaccine development.

## 2. Material and Methods

### 2.1. Study Site and Study Population

The study was conducted in two community clinics, Saponé Marché and Kounda located in the Saponé Health District, approximately 50 km southwest of Ouagadougou, the capital of Burkina Faso. The Saponé Health District is located at 12°13′N, 1°48′W and has approximately 80,000 inhabitants, most of them (>95%) belonging to the Mossi ethnic group. Malaria transmission is perennial and seasonal in this district and is characterized by a peak during the rainy season (May–October). The entomologic inoculation rate was estimated at 200 infective bites/person/year in the study area [17]. The incidence of clinical malaria cases in children less than five years of age was estimated at 1.3 episodes/child-year [18].

About 500 children under the age of five living in the two communities were targeted for this study.

During the follow-up period, all malaria cases were managed by the study clinical staff with no cost. Clinical management was based on the National Policy guidelines, which consisted of artemisinin-based combination therapy (Coartem; Roche, Basel, Switzerland) for uncomplicated malaria cases and quinine salt for severe malaria cases.

### 2.2. Ethical Considerations

The study was part of a large epidemiological study aimed at assessing malaria morbidity and mortality in children under the age of five years (DMID protocol 06-0020). The study protocol was reviewed and approved by Office of Clinical Research Affairs (OCRA) within the Division of Microbiology and Infectious Disease (DMID at National Institute of Allergy and Infectious Diseases (NIAID) of the USA). It was also approved by the Health Research Ethics Review Committee in Burkina Faso.

A clear description of the study and all anticipated risks and benefits was explained to the participants before a written informed consent was voluntarily obtained from the participants or their legal guardians.

### 2.3. Sample Collection

Blood samples were obtained by finger prick from children aged 3–59 months. Thick and thin smears and blood spotted on filter paper (Whatman No. 2) were collected during two cross-sectional surveys conducted in February and September 2007, respectively, dry and rainy season time points, for asymptomatic cases detection. Children were then followed up during one year at their relevant community clinics for malaria symptomatic cases detection.

Thick and thin smears were stained with Giemsa and examined under light microscope for malaria parasites identification and counting.

The spotted filter paper was kept at room temperature with silica gel desiccant prior to genotyping analysis.

For the purpose of this study, the following definitions were adopted: symptomatic malaria case was defined as children with axillary temperature ≥37.5°C bearing asexual* P. falciparum* parasite irrespective of parasite density; asymptomatic malaria case was defined as the presence of asexual* P. falciparum* parasite irrespective of parasite density, in the absence of clinical symptoms.

### 2.4. Microscopic Examination

Thick and thin blood smears were stained with Giemsa and microscopically examined to identify monoinfections with* P. falciparum*. Asexual* P. falciparum* parasites were estimated against 200 leucocytes and the parasite density was calculated based on a mean of 8000 leucocytes per microliter of blood.

### 2.5. *Plasmodium Falciparum* DNA Extraction

Parasite DNA was extracted from blood spots using QiAmp DNA blood mini kit according to the manufacturer's protocol (Qiagen; Valencia, CA). DNA samples were aliquoted in sterile tubes and stored at −20°C until polymerase chain reaction (PCR) amplification.

### 2.6. *Eba-175*,* msp-3*, and* ama-1* Fragments Amplification

*Eba-175* genotyping was performed by nested PCR as described elsewhere [4]. The primers sequences and the amplification reaction conditions are described in Table 1. A seminested PCR was used to amplify the polymorphic region of* msp-3* gene (see Table 1). This region is predominantly confined to the N-terminal extremity within the heptad-repeat previously identified as a site of antigenic diversity among MSP-3 polypeptides [19]. Briefly, two rounds of PCR amplification were performed. The first-round PCR amplification was achieved with 5 *µ*L of DNA template in a final volume of 25 *µ*L. The second one was performed similarly to the first with 2.5 *µ*L of the first PCR product being DNA template. Each PCR round amplification contained 1 *µ*M of each primer, 1 unit of Taq DNA polymerase (Invitrogen, Carlsbad, CA), 2.5 *µ*L of 10x PCR buffer (Invitrogen), 2.0 mM MgCl_2_, and 200 *µ*M dNTP (Invitrogen). The PCR cycling conditions for the first and the second reaction are described in Table 1.

The first PCR reaction was similar to the above* msp-3* first amplification reaction using a set of primers (VM785/3 and VM990) indicated in Table 1. The second-round reaction mixtures for* ama-1* were performed in a final volume of 50 *µ*L that contained 5 *µ*L of 10x PCR buffer (Invitrogen) and 2 units of Taq DNA polymerase with 1 *µ*M each of the* ama-1* VM815 and VM990 primers. The PCR products from the* ama-1* second reaction were digested by using three restriction enzymes* Mse* I,* Ssp* I, and* BfCU* I, which generated fragments whose size corresponded to the 3D7, K1, and HB3/7G8* ama-1* allele classes, respectively.

### 2.7. Internal Quality Control and Data Interpretation

Purified DNA from* P. falciparum* 3D7 (MRA-102G), HB3-B2 (MRA-149G), and K1 (MRA-159) was provided by the Malaria Research and Reference Reagent Resources Center, American Type Culture Collection (Manassas, VA), and used as positive controls during the PCR amplification. The positive controls were used to score and interpret fragment sizes.

The* Eba-175* alleles (CAMP and FCR3) were identified as single fragments, approximately 714 and 795 base pairs in length, respectively. The* msp-3* K1 and 3D7 alleles were identified as single fragments, approximately 500 and 400 base pairs in length, respectively. All other* msp-3* fragments of different sizes were also reported. Finally, the* ama-1*-K1,* ama-1*-3D7, and* ama-1*-HB3/7G8 alleles were identified as single fragments of 285, 400, and 335 base pairs in length, respectively, when subjected to digestion with* Mse* I,* Ssp* I, and* BfCU* I, respectively.

Mixed infections were defined as the simultaneous presence of K1 and 3D7 msp-3 alleles or the F and C* eba-175* fragments or the presence of two or three of the* ama-1* fragments within the same sample.

### 2.8. Statistical Analysis

The allelic data input was double entries in Excel format and was analyzed using the statistical software Epi Info v6.04a (http://www.cdc.gov/epiinfo/Epi6/EI6dnjp.htm). This software was used to obtain the carrier frequencies of the different alleles. Individual allele frequencies were estimated using GenAlEx 6.5 [20].

Inclusion in the combined dataset was limited to samples with missing data for no more than one marker.

In order to assess if the prevalence of* msp-3*,* eba-175*, and* ama-1* alleles varies with age, the data were categorized into three age groups (≤1 year, 1 to 3 years, and >3 years) based on the difference in the development of acquired immunity.

The chi-square and Fisher's exact tests were used for data analysis with *p* < 0.05 value used as statistical significance threshold end point.

Genetic diversity was estimated by determining the heterozygosity based on the three antigens combined, where heterozygosity (*h*) = 1 − ∑*pi* ^2^ ranged from 0 to 1, where *pi* is the frequency of the *i*th allele. The *t*-test was used to compare mean heterozygosity between the different groups.

## 3. Results

A total of 427 children with a mean age of 2.2 years (95% confidence interval (CI), 2.1–2.4) were included in this study. The genetic analyses included 427 samples representing 228 and 199 samples collected, respectively, from asymptomatic and symptomatic malaria children. This genetic analysis included only samples which were microscopically known positive for* Plasmodium falciparum* parasite and therefore successfully scored for at least one of the three genes.

### 3.1. Allele Prevalence in Symptomatic and Asymptomatic Malaria Cases

We investigated whether asymptomatic and symptomatic malaria patients could have differences in the prevalence of the* eba*-175 alleles. Genotyping analysis revealed that the frequency of the FCR3 allele was significantly higher in asymptomatic infections compared to the symptomatic infections (*p* = 0.0001). The* ama-1-*HB3 allele was also overrepresented in the asymptomatic infections, but without reaching a statistically significant difference (Table 2). However, the* msp-3_K1* allele showed a significant prevalence in symptomatic carriers when compared to asymptomatic carriers (*p* = 0.00001) (Table 2).

A comparison of the allele's prevalence within each group (asymptomatic or symptomatic) revealed that the* ama-1_HB3* allele and the* eba-175*_*FCR3* allele were the most prevalent ones in both groups. The* msp-3_3D7* was the most common* msp-3* alleles in the asymptomatic group.

The analysis of the frequency of the individual alleles showed that* eba-175 FCR3* and the* msp-3 K1* alleles were more common in asymptomatic versus symptomatic group and that the difference was significant (Table 2).

### 3.2. The Effect of Age on* msp-3*,* ama-1*, and* eba-175* Allele Frequency

In order to assess if the prevalence of* msp-3*,* eba-175*, and* ama-1* alleles varies with age, the data were categorized into three age groups (≤1 year, 1 to 3 years, and >3 years) based on the difference in the development of acquired immunity. Statistical analysis of the distribution of the different alleles by age demonstrated some structure in the allele frequencies. The distribution of* msp-3* alleles showed a higher and statistically significant prevalence of* msp-3 3D7* compared to* msp-3 K1* alleles in all age groups irrespective of the clinical features, except in the “1–3 years” category where a statistically significant lower frequency (*p* = 0.005) of* msp-3* 3D7 alleles was observed (Table 3).

The analysis of the* ama-1* alleles showed that even the HB3 allele was the most frequent one across all age categories, the difference was statistically significant only for children <1 and >3 years of age in the symptomatic group (Table 3).

A higher prevalence of* eba-175_FCR3* alleles compared to* CAMP* was notified in all age categories, except in symptomatic children aged >3 years (Table 3). The difference was statistically significant only in the children aged between 1–3 years of age in both group (*p* < 0.5).

## 4. Discussion

The present study was designed to analyze the allelic diversity of the three main malaria vaccine candidates in symptomatic and asymptomatic malaria infected children living in the southern region of Burkina Faso. The study was focused on domain I of AMA-1 representing the most polymorphic region of the gene [13, 16, 21] and known as a major target of anti-AMA-1 protective antibodies [22]. The N-terminal extremity of the* msp-3* gene within and flanking the heptad-repeat selected for this genetic analysis was identified in previous study as the site of antigen diversity among MSP-3 polypeptides [19]. Finally, the polymorphic region of* eba-175* subject to the present genetic analysis was the exon I located in region II which is known to code for a repeated sequence of two Duffy binding-like domains (F1 and F2). The two domains F1 and F2 are being separated by highly divergent dimorphic region III, through the insertion of either a 342-base pair segment in FCR-3 strains (F-loop) or a 423-base pair segment in CAMP strains (C-loop).

The genetic diversity influence of such known surface antigens in malaria pathogenesis was discussed in various previous studies generating conclusions with a need for further investigations in different malaria setting. Indeed, the importance of the relationship between* eba1-175* dimorphism and malaria clinical status was demonstrated in children living in central Africa and specifically from Gabon [12, 23].

The importance of AMA-1 as a malaria vaccine candidate provides further justification for exploring the role of genetic diversity in this antigen. A strong imbalance in* ama-1* genetic diversity between symptomatic and asymptomatic infections was highlighted in a previous study [24] suggesting that malaria morbidity may be strain specific.

The results from this present study confirmed that the* eba-175* gene was dimorphic with F and C alleles present in general prevalence as shown in previous studies [25–27]. While the F alleles were predominant, the results also showed that there was no relationship with the clinical status of the patients (asymptomatic and symptomatic). However, the study showed a high proportion of mixed segment infection, including C and F alleles significantly in asymptomatic cases. This finding is in line with previous reports in Asia [28] and in Africa [29].

The analysis of* ama-1* alleles in our study showed lack of association in genotype prevalence with malaria infection contrasting the finding from previous studies. Strong imbalance between symptomatic and asymptomatic infections was found in the distribution of residues at several clustered polymorphic positions of* ama-1* genotypes [24]. However, the* ama-1*_HB3/7G8 genotypes were overrepresented in the study area in both symptomatic and asymptomatic groups compared to the other genotypes (K1 and 3D7). The common PCR method used in this study does not provide enough resolution to discern individual point mutations in the domain I predicted to arise from diversifying selection due to a protective immune response [13, 30–32]. Direct sequencing of the* ama-1* gene product would probably better address the role of such polymorphism in the pathogenesis of malaria.

The* msp-3_K1* alleles were associated (*p* < 0.0001) with higher risk of clinical malaria in Table 2. However, this association remained only statistically significant in the symptomatic population between 1 year of age and 3 years of age but not in the other ages (groups <1 year and >3 years). In parallel, the presence of the* msp-3_3D7* alleles was associated with asymptomatic malaria only in the age group of 1–3 years. These findings highlight the relevance of analyzing the polymorphism of the* P. falciparum* parasite based on subclasses with closer interval of age instead of large range of age. Previous studies analyzing the age-allele specific immunity constitute a perfect illustration of that complexity of malaria antigen polymorphism [33] in which polymorphism is a common mechanism for immune evasion used by the parasite [34].

The PCR-RFLP is use as a technical approach to analyze the allelic diversity in this study. Even though this is a useful approach based analysis, inexpensive and ease to determine the number of different parasite genotypes infecting an individual patient or in assessing the multiplicity of infection (MOI), the approach is of limited utility for interpreting some patterns as the size itself (principle of polymorphism) may be under selection [35, 36] or for identifying the exact variation in the event that several polymorphisms affect the same restriction enzyme recognition site [37].

## 5. Conclusion

This comparative analysis of the role of allelic polymorphism of the* eba-175*,* msp-3*, and* ama-1* gene in* P. falciparum* isolates from asymptomatic and symptomatic malaria cases indicated that the polymorphism was comparable between asymptomatic and symptomatic malaria groups in our study area. Except for few differences observed in the* eba-175* and* msp-3* alleles frequencies, the overall polymorphism of the three genes together did not show any alleles association with symptomatic or asymptomatic malaria. Nevertheless, these findings give broad insights about the general distribution of the* P. falciparum* polymorphism for the development of malaria vaccine trial sites. Our results highlighted the need of using more advanced techniques such as DNA sequencing in order to shed light on this relevant debate.

## Figures and Tables

**Table 1:** Sequences of the primers and PCR conditions.

	Sequences	PCR conditions^*∗*^
	*eba-175*	
First reaction	5′-CAAGAAGCAGTTCCTGAGGAA-3′ (forward) 5′-CTCAACATTCATATTAACAATTC-3′ (reverse)	D (94°C for 1 min), A (56°C for 1 min), Ex (72° for 2 min), FEx (72°C for 3 min), Cy (29 cycles)
Second reaction	5′-GAGGAAAACACTGAAATAGCACAC-3′ (forward) 5′ CAATTCCTCCAGACTGTTGAACAT-3 (reverse)	D (94°C for 1 min), A (56°C for 1 min), Ex (72°C for 2 min), FEx (72°C for 3 min), Cy (24 cycles)
	*ama*-*1*	
First reaction	VM785/3: 5′-CCGGATCCCCTTTGAGTTTACATATATG-3′ (forward) VM990: 5′-AAA TTC TTT CTA GGG CAA AC-3′ (reverse)	D (95°C for 2 min 30 s), A (51°C for 30 s), Ex (68°C for 45 s), FEx (68°C for 5 min), Cy (30 cycles)
Second reaction	VM815/3: 5′-GGA ACT CAA TAT AGA CTT CC-3′ (forward) VM990: 5′-AAA TTC TTT CTA GGG CAA AC-3′ (reverse)	D (95°C for 2 min 30 s), A (51°C for 30 s), Ex (68°C for 45 s), FEx (68°C for 5 min), Cy (35 cycles)
	*msp*-*3*	
First PCR reaction	*159F*: 5′-ATGTTGCTAGTAAAGAAATTG-3′ (forward) *745R*: 5′-CATAACTAGAAGCTTCTTTTGC-3′ (reverse)	D (94°C for 30 s), A (54°C for 30 s), Ex (68°C for 2 min), FEx (68°C for 5 min), Cy (30 cycles)
Second PCR reaction	*188F*: 5′-ATAATCTTAACTTAAGAAATGC-3′ (forward) *745R*: 5′-CATAACTAGAAGCTTCTTTTGC-3′ (reverse)	D (94°C for 30 s), A (54°C for 30 s), Ex (68°C for 2 min), FEx (68°C for 5 min), Cy (40 cycles)

^*∗*^D: denaturation; A: annealing; Ex: extension: FEx: final extension; Cy: number of cycles.

**Table 2:** Prevalence of *ama-1*, *eba-175*, and *msp-3* genotypes from symptomatic and asymptomatic malaria cases.

Alleles	Asymptomatic		Symptomatic		*p* value (between groups)
	No (%)	*h*	No (%)	*h*	
*Ama-1*_K1	75 (33.3)	0.44	39 (32.8)	0.44	0.9
*Ama-1*_3D7	78 (34.7)	0.45	43 (33.3)	0.44	0.8
*Ama-1*_HB3/7G8	131 (58.5)	0.48	55 (47.8)	0.50	0.06
*p* value (within group)	≪0.0001^*∗*^		0.03^*∗*^		
*Eba-175*_FCR3	202 (88.6)	0.50	95 (67.9)	0.46	0.0001^*∗*^
*Eba-175*_CAMP	107 (46.9)	0.20	51 (36.4)	0.44	0.05^*∗*^
*p* value (within group)	≪0.0001^*∗*^		≪0.0001^*∗*^		
*Msp3*_K1	83 (36.4)	0.46	103 (59.5)	0.49	0.00001^*∗*^
*Msp3*_3D7	115 (50.4)	0.50	98 (56.32)	0.49	0.2
*p* value (within group)	0.002^*∗*^		0.5		

*h*: heterozygosity.

^*∗*^Significant *p* value <0.05.

**Table 3:** Age variation of *eba-175*, *msp-3*, and *ama*-*1* allele prevalence.

	Alleles	Asymptomatic malaria cases			Symptomatic malaria cases		
		≤1 year (45)	1–3 years (99)	>3 years (84)	≤1 year (66)	1–3 years (92)	>3 years (41)
*eba*-*175*	EBA-175_CAMP	46.7	52.5	40.5	30.3	17.4	36.6
	EBA-175_FCR	95.6	87.9	85.7	48.5	48.9	43.9
	*p* value	*0.4*	*0.01* ^*∗*^	*0.12*	*0.08*	*0.005* ^*∗*^	*0.4*

*ama-1*	AMA-1_K1	37.8	38.4	23.8	12.1	21.7	26.8
	AMA-1_3D7	37.8	38.4	27.4	15.2	30.4	12.2
	AMA-1_HB3	66.7	49.5	61.9	22.7	29.3	31.7
	*p* value	*0.006* ^*∗*^	*0.18*	*0.00001* ^*∗*^	*0.24*	*0.35*	*0.1*

*msp-3*	MSP-3_K1	42.2	34.3	35.7	45.5	59.8	43.9
	MSP-3_3D7	51.1	52.5	47.6	60.6	39.1	53.7
	*p* value	0.00001^*∗*^	0.00001^*∗*^	0.00001^*∗*^	0.03^*∗*^	0.00001^*∗*^	0.5

^*∗*^Significant *p* value <0.05.

## Abbreviations

*P. falciparum*: * Plasmodium falciparum*
PCR: Polymerase chain reaction
DNA: Deoxyribonucleic acid
AMA-1: Apical membrane antigen-1
EBA-175: Erythrocyte binding antigen-175
MSP-3: Merozoite surface protein-3
PCoA: Principal coordinates analysis
ACT: Artemisinin combination therapy
WHO: World Health Organization
DMID: Department of Microbiology and Infectious Diseases
ASY: Asymptomatic
SYM: Symptomatic.

## References

[1] WHO (2014). *World Malaria Report*.

[2] Gupta S., Hill A. V. S., Kwiatkowski D., Greenwood A. M., Greenwood B. M., Day K. P. (1994). Parasite virulence and disease patterns in *Plasmodium falciparum* malaria. *Proceedings of the National Academy of Sciences of the United States of America*.

[3] Soulama I., Sawadogo M., Nebie I. (2006). Genetic diversity of *P. falciparum* and pathogenesis of the severe malarial anaemia in children under 5 years old in the province of Boulgou, Burkina Faso. *Bulletin de la Societe de Pathologie Exotique*.

[4] Soulama I., Nébié I., Ouédraogo A. (2009). *Plasmodium falciparum* genotypes diversity in symptomatic malaria of children living in an urban and a rural setting in Burkina Faso. *Malaria Journal*.

[5] Camus D., Hadley T. J. (1985). A Plasmodium falciparum antigen that binds to host erythrocytes and merozoites. *Science*.

[6] Klotz F. W., Orlandi P. A., Reuter G. (1992). Binding of *Plasmodium falciparum* 175-kilodalton erythrocyte binding antigen and invasion of murine erythrocytes requires *N*-acetylneuraminic acid but not its *O*-acetylated form. *Molecular and Biochemical Parasitology*.

[7] Blackman M. J., Scott-Finnigan T. J., Shai S., Holder A. A. (1994). Antibodies inhibit the protease-mediated processing of a malaria merozoite surface protein. *The Journal of Experimental Medicine*.

[8] Silvie O., Franetich J.-F., Charrin S. (2004). A role for apical membrane antigen 1 during invasion of hepatocytes by *Plasmodium falciparum* sporozoites. *The Journal of Biological Chemistry*.

[9] Meraldi V., Nebié I., Tiono A. B. (2004). Natural antibody response to *Plasmodium falciparum* Exp-1, MSP-3 and GLURP long synthetic peptides and association with protection. *Parasite Immunology*.

[10] Soe S., Theisen M., Roussilhon C., Khin-Saw-Aye, Druilhe P. (2004). Association between protection against clinical malaria and antibodies to merozoite surface antigens in an area of hyperendemicity in Myanmar: complementarity between responses to merozoite surface protein 3 and the 220-kilodalton glutamate-rich protein. *Infection and Immunity*.

[11] Thomas A. W., Trape J.-F., Rogier C., Goncalves A., Rosario V. E., Narum D. L. (1994). High prevalence of natural antibodies against *Plasmodium falciparum* 83- kilodalton apical membrane antigen (PF83/AMA-1) as detected by capture- enzyme-linked immunosorbent assay using full-length baculovirus recombinant PF83/AMA-1. *American Journal of Tropical Medicine and Hygiene*.

[12] Cramer J. P., Mockenhaupt F. P., Möhl I. (2004). Allelic dimorphism of the erythocyte binding antigen-175 (*eba*-175) gene of *Plasmodium falciparum* and severe malaria: significant association of the C-fragment with fatal outcome in Ghanaian children. *Malaria Journal*.

[13] Policy S. D., Conway D. J. (2001). Strong diversifying selection on domains of the *Plasmodium falciparum* apical membrane antigen 1 gene. *Genetics*.

[14] Huber W., Felger I., Matile H., Lipps H. J., Steiger S., Beck H.-P. (1997). Limited sequence polymorphism in the *Plasmodium falciparum* merozoite surface protein 3. *Molecular and Biochemical Parasitology*.

[15] Garg S., Alam M. T., Das M. K. (2007). Sequence diversity and natural selection at domain I of the apical membrane antigen 1 among Indian *Plasmodium falciparum* populations. *Malaria Journal*.

[16] Marshall V. M., Zhang L., Anders R. F., Coppel R. L. (1996). Diversity of the vaccine candidate AMA-1 of *Plasmodium falciparum*. *Molecular and Biochemical Parasitology*.

[17] Nebie I., Diarra A., Ouedraogo A. (2008). Humoral responses to *Plasmodium falciparum* blood-stage antigens and association with incidence of clinical malaria in children living in an area of seasonal malaria transmission in Burkina Faso, West Africa. *Infection and Immunity*.

[18] Ouédraogo A., Tiono A. B., Diarra A. (2013). Malaria morbidity in high and seasonal malaria transmission area of Burkina Faso. *PLoS ONE*.

[19] McColl D. J., Anders R. F. (1997). Conservation of structural motifs and antigenic diversity in the *Plasmodium falciparum* merozoite surface protein-3 (MSP-3). *Molecular and Biochemical Parasitology*.

[20] Peakall R., Smouse P. E. (2012). GenAlEx 6.5: genetic analysis in Excel. Population genetic software for teaching and research—an update. *Bioinformatics*.

[21] Escalante A. A., Grebert H. M., Chaiyaroj S. C. (2001). Polymorphism in the gene encoding the apical membrane antigen-1 (AMA-1) of *Plasmodium falciparum*. X. Asembo Bay Cohort project. *Molecular and Biochemical Parasitology*.

[22] Hodder A. N., Crewther P. E., Anders R. F. (2001). Specificity of the protective antibody response to apical membrane antigen 1. *Infection and Immunity*.

[23] Touré F. S., Bisseye C., Mavoungou E. (2006). Imbalanced distribution of *Plasmodium falciparum* EBA-175 genotypes related to clinical status in children from Bakoumba, Gabon. *Clinical Medicine and Research*.

[24] Cortés A., Mellombo M., Mueller I., Benet A., Reeder J. C., Anders R. F. (2003). Geographical structure of diversity and differences between symptomatic and asymptomatic infections for *Plasmodium falciparum* vaccine candidate AMA1. *Infection and Immunity*.

[25] Orlandi P. A., Kim Lee Sim B., Chulay J. D., Haynes J. D. (1990). Characterization of the 175-kilodalton erythrocyte binding antigen of *Plasmodium falciparum*. *Molecular and Biochemical Parasitology*.

[26] Ware L. A., Kain K. C., Lee Sim B. K., Haynes J. D., Baird J. K., Lanar D. E. (1993). Two alleles of the 175-kilodalton *Plasmodium falciparum* erythrocyte binding antigen. *Molecular and Biochemical Parasitology*.

[27] Duraisingh M. T., Maier A. G., Triglia T., Cowman A. F. (2003). Erythrocyte-binding antigen 175 mediates invasion in *Plasmodium falciparum* utilizing sialic acid-dependent and -independent pathways. *Proceedings of the National Academy of Sciences of the United States of America*.

[28] Sorontou Y., Pakpahan A. (2012). Allele dimorphic of Eba-175 gene of *Plasmodium falciparum* and associated with clinical manifestation in Jayapura District, Papua, Indonesia. *Asian Transactions on Basic and Applied Sciences*.

[29] Touré F. S., Mavoungou E., Ndong J. M. M., Tshipamba P., Deloron P. (2001). Short communication: erythrocyte binding antigen (EBA-175) of *Plasmodium falciparum*: improved genotype determination by nested polymerase chain reaction. *Tropical Medicine and International Health*.

[30] Ouattara A., Mu J., Takala-Harrison S. (2010). Lack of allele-specific efficacy of a bivalent AMA1 malaria vaccine. *Malaria Journal*.

[31] Escalante A. A., Lal A. A., Ayala F. J. (1998). Genetic polymorphism and natural selection in the malaria parasite *Plasmodium falciparum*. *Genetics*.

[32] Verra F., Hughes A. L. (2000). Evidence for ancient balanced polymorphism at the Apical Membrane Antigen-1 (AMA-1) locus of *Plasmodium falciparum*. *Molecular and Biochemical Parasitology*.

[33] Osier F. H. A., Polley S. D., Mwangi T., Lowe B., Conway D. J., Marsh K. (2007). Naturally acquired antibodies to polymorphic and conserved epitopes of *Plasmodium falciparum* merozoite surface protein 3. *Parasite Immunology*.

[34] Terheggen U., Drew D. R., Hodder A. N. (2014). Limited antigenic diversity of *Plasmodium falciparum* apical membrane antigen 1 supports the development of effective multi-allele vaccines. *BMC Medicine*.

[35] Takala S. L., Escalante A. A., Branch O. H. (2006). Genetic diversity in the Block 2 region of the merozoite surface protein 1 (MSP-1) of *Plasmodium falciparum*: additional complexity and selection and convergence in fragment size polymorphism. *Infection, Genetics and Evolution*.

[36] Rice B. L., Acosta M. M., Pacheco M. A., Escalante A. A. (2013). Merozoite surface protein-3 alpha as a genetic marker for epidemiologic studies in *Plasmodium vivax*: a cautionary note. *Malaria Journal*.

[37] Rasmussen H. B., Magdeldin S. (2012). Restriction fragment length polymorphism analysis of PCR-amplified fragments (PCR-RFLP) and gel electrophoresis—valuable tool for genotyping and genetic fingerprinting. *Gel Electrophoresis—Principles and Basics*.

